# Report of the Medical Department of the University of Pennsylvania, for the Session of 1851—to the Alumni of the School

**Published:** 1852-11

**Authors:** 


Art. VI.—*Report of the Medical Dipartment of the University of Pennsylvania, for the session of* 1851–2 *to the alumni of the school.* By the Medical Faculty.

There are some curious features in the document, the tiile of which we have given above, that naturally challenge the attention of the medical profession. The American Medical Association has suggested, as a measure of reform in medical teaching, the prolongation of the lecture terms of the schools to six months, and the medical department of the University of Pennsylvania leaped into the measure with a celerity that bordered on bustling. Year after year she made the air sonorous with her bugle notes on the subject, and from her importunities to the American Medical Association, she seemed to have taken up the idea that the sole object of the existence of that body was to hold up the hands of the good old Moses of medical reform, in the battles with the Ammonites. She constantly occupied the annual meetings of the Association with her bills of charges for the great sacrifices she had made in furthering the suggestions of the Association for reform in teaching.

But a change has come over the sturdy champion of long terms. The circular recently issued by the faculty, of what is known as the old school of Philadelphia, thus announces her position in the arena of reform and competition

"It is well known to the friends of this school that it has been engaged in an earnest effort to improve the system of medical education in the United States. One of the means by which it has sought to accomplish this object has been the prolongation of the scholastic term of instruction."

The reader must remember now that in our quotation the object and motive which led to the prolongation of the lecture term are set forth, and that the claim is urged that the object was exclusively the improvement of the system of medical education in the United States. It is very important that this shall be remembered, for, if forgotten, the reader cannot appreciate the curious attitude that "circumstances," as potent sometimes in medical as in political circles, have forced the University of Pennsylvania to take.

In 1849–50 the term of that school had been extended to six months. By some this prolongation was looked upon as a vigorous stride towards improvement. Others took a reverse view, and we were among the number.—The Harvard school opposed the extension, and based her opposition on ample reasons.

But the school of the University of Pennsylvania claimed that she made the prolongation in obedience to an earnest wish to improve the system of medical instruction in the United States. She annually filled the ears of the profession with testimonials of the gratification she experienced from this great reform. Her evidence to its vast merits was unqualified. But, at the same time, her faculty announced that they were quite aware of the risk incurred; but as no improvement could be expected unless some school would venture to take the first step, "ours, as the oldest, and among the most prominent, seemed especially called on to set the example." Thus it was that a sense of duty pushed the school towards the fires of martyrdom, and as the first effects were found to be "excellent," in the estimation of the faculty, that body urged the general adoption of the prolongation with a vehemence quite worthy of the holy cause. Even in the present document the faculty remark that they are gratified in being able to say, as they do with perfect sincerity, that "there has been a progressive improvement in all respects, increasing steadiness of deportment, attention to study, and earnestness of self-improvement have been observable; and the examinations, though not less strict than formerly, have each year resulted in relatively fewer failures to attain the honors of the Institution." Here then we have a most satisfactory result of reform; the object of the reform being the improvement of Medical Pupils, seems to have been fully realised by the University of Pennsylvania. The Faculty had felt that their Institution was a city set on a hill, whose example was essential, and whose light should not be hid. They felt that they were adventuring, but were encouraged by duty; and according to the Faculty, the great reform answered *every end* that had been promised for it, by those who suggested it. Of course the reader is prepared to hear that the old and prominent Institution is persevering in the good works that brought forth a plentiful crop of excellent fruit. But, alas! although the classes improved in their steadiness, excellence, earnestness in study, and in capacity for standing examinations for the honors of the school; although everything worked well, there was a debenture; the classes began to fall off. The reform had worked admirably in qualifying students for the doctorate, and for practice, but the Professors found that their fees were diminishing, and after all the "earnest efforts to improve the system of Medical education in the United States," after proclaiming year by year that the "earnest efforts" were perfectly successful, in the very midst of these elevating and glowing prospects, the Faculty announce that they cannot stand the benefits of the reform any longer. The reform was a good thing to write about, to talk about, to trumpet over the earth, to bring annually before the American Association; it accomplished everything that had been promised for it, it improved students most essentially, made better Doctors, thereby offering something to society, for low per centage in some of the columns of vital statistics, but, notwithstanding all these things, the Faculty had discovered that it diminished, not a fair and reasonable compensation, but to a small extent an extravagant income, if compared with the revenue of a large majority of the Medical Schools of the United States, and on account of this diminution, the Faculty turn their backs upon that reform, and its good results, which they had lauded annually.

After all the " encouraging results " that have been ardently and perseveringly pressed upon the profession for years, as the fruits of six months terms of lectures; the reader is scarcely prepared to hear that the Faculty have determined to shorten the " good time " to a five months term! The Faculty do not pretend that they have changed their views of the excellence of what they deem the essential results of the long term. But what? Why, the fees are diminishing. At first the classes increased, and then the reform was magnificent; now the classes are lessening, and though the reform was, and is, according to the circular, beneficial to the medical students, to the profession, and to society; yet the Faculty are done with it. The first draught made at extension, caught 509 matriculants, the second 499, third 439, fourth 466, and for 1851–'52, only 410. The class of 1851–'52 opened the eyes of the Faculty to the enormity of reform. The age and prominence of the school induced the display of a love of reform, but " prominence " and long columns of matriculants being matters of some moment to the school; as soon as shadows seem to fall on them, the reform is hurried overboard with hot haste.—What if students were improved, what if the graduates under the long term were better qualified to practice medicine than the graduates under the former system; what if society had its chances of living increased, was the income of the Professors nothing? Could they be expected to benefit the profession and the community on a class of four hundred and ten matriculants? The short class shortened the term, and whether the intention is to continue to shorten the term, if the classes keep up the unpropitious feature of growing less by degrees, we are not informed. It was hoped, by the Faculty, that they would force other schools to follow in their wake, and that the classes would increase; in both these there was a sad mistake, and the cry is, now, perish reform and all its works.

If it were not for their connection with medical dignity and honor, the efforts of the Faculty to relieve themselves from, what they evidently consider a natural suspicion, that their devotions to Medical Science, and "earnest efforts to improve the system of medical education," are more influenced by the effect of the "efforts" on their income, than by the good results of reform, would be infinitely amusing. The "efforts" were "earnest," indeed, as long as the class increased, and the Faculty admit now, that the results to students, and to society were satisfactory. It is a plain matter of fact, that the Faculty did not feel inclined to submit to any sacrifice, in advancing the interests of the profession. The interests of the Profession and of the Professors, must run *pari passu.*

We do not object to the changes in the system of managing the affairs of the Medical department of the University of Pennsylvania. What we object to is, to see the Faculty strutting through the land with very broad phylacteries, covered with texts of scripture of exceeding devoutness. All men cannot, at will assume airs of disinterestedness for an emergency, and we do not like to see those who can, too lavish of the display. If the circular of the Faculty had fairly stated the fact, that the University was favorable to Medical reform as long as that reform swelled the income of the Professors, and that in spite of the admitted great benefits of the reform, to the profession, and to society, the Faculty deserted it, when their classes began to diminish in number; no one could have objected to the exercise of the right of the Faculty to make such a public announcement. It is true, there might have been cavillers who would have said that a class of four hundred students should be sufficient to gratify any pride of prominence, and any reasonable desires of income. Some might have gone farther, and expressed the belief that a class of that size should have been sufficient to keep a Medical Faculty from deserting a good cause that was working well. The "prominent school" of the country was scarcely justified in withdrawing from a fair test of a new educational scheme, for no better reason, than because matriculants were diminishing in numbers to some extent. It is a sorry lesson to Medical schools, whose best emoluments are far below the liberal scale that frightened the Faculty of the University of Pennsylvania. These schools find in the indisposition of the Faculty of that Institution, to jeopard in the least degree their emoluments in favor of a reform that they said worked admirably, an excuse for not making any "earnest efforts" to improve themselves, their pupils, or the community. Alas! for science and philanthropy.

The evidence of this position of the Faculty of the University of Pennsylvania, given by their own circular is as clear as the noon-day sun. We cannot believe that they hold the intelligence of Medical readers in as low an estimate as they indicate, in presuming that educated minds were to be deceived by the specious ethics in which the retreat from the good work of reform is attempted to be concealed. The sleeping draught was made so strong, that the narcotic is easily smelled. But the reader may incredulously ask, can these things be? Are you not giving us distorted inferences, instead of facts? Let the doubter read the following extracts from the circular:

"There are two distinct principles upon which a medical school may be conducted; one having reference to the interests of the institution, the medical profession, and the country; the other to the temporary and pecuniary interests of the professors.

If the first of these principles be acted on, the efforts of the school will be so directed as to secure the highest possible qualifications in the graduates. Not only will the amplest opportunities for instruction be afforded, but rules will be established, and, as far as practicable, carried into effect, for securing to the pupil the full advantage of these opportunities. Certain prerequisites to graduation will be announced, upon the basis of which the public may estimate the value of the degree; and, in order that the public may not be deceived, these prerequisites will be religiously adhered to, unless in a few exceptional cases, in which their strict enforcement might work great individual hardship.

The operations of a school based upon the second principle referred to, that, namely, which regards the temporary and pecuniary interests of the professors as the main object, it is unnecessary to follow out in their details.—Of course, they will be directed to the securing of the greatest practicable amount of income; and all measures will be deemed legitimate which are admissible in the struggles of mercantile competition.

The Faculty of the University need not say that they acknowledge fealty to the first of these principles. Independently of the obligations of common honor and honesty, they consider themselves the depositaries of a great trust, in the proper management of which their personal credit is involved. The school with which they are charged has come to them with a reputation, based upon a long course of honorable and successful effort on the part of their predecessors. They consider it a duty to maintain this reputation by all the means within their reach, even at the risk of loss to themselves individually. They dare not compromise the character of the school by lowering its requisitions and relaxing its rules, though by doing so they might possibly for a time increase the number of matriculants, and of course their pecuniary income. They have felt themselves bound, so far as might be in their power, to keep the business of medical instruction in equal advance with the other great national interests. This could be done only by taking an occasional forward step, so as to test the capabilities of the country. It does not follow that such an experiment must always be successful. Should it on trial prove to have been premature, no pride of consistency should prevent a retracing of the step, until it is ascertained at what point a firm stand may be taken, in accordance with the best interests of the school and the profession. The size of the classes will always enter somewhat into the estimate of the standing of a school; and a certain number of pupils is, for obvious reasons, essential to its efficient operation, under a system by which such institutions support themselves. While, therefore, aiming at improving the character of the classes, the Faculty must not be indifferent to their numbers. As before stated, they are happy in the conviction that, in the former of these respects, there has been for several years a gradual improvement under the improved arrangements of the school. But it must also be admitted that, under the same arrangements, with the opposing influence of a powerful competition, the numbers have not been of late altogether satisfactorily maintained; and there is some reason to apprehend that, without change, there may be a still further decrease."

That language is certainly plain enough. We feel as though Horace was thinking of something of the kind when he wrote:

I know the right, and I approve it too;
I hate the wrong, and yet the wrong pursue,"

Amidst all the flaunting of the "considerations by which the Faculty were influenced;" is anything more conspicuous in bringing about "certain modifications of the recent course," than that which relates to the "temporary and pecuniary interests of the Professors." While we read the flaming zeal for "the first of the principles," for the management of Medical Schools, we feel that here is the proper spirit. We enjoy the sly thrusts made at those "operations of a school based" on that "which regards the temporary and pecuniary interest of the Professors, as the main object," and all seemed so overflowing with high toned Medical honor and zeal for the interest alike, of the profession and of humanity, that we were about to throw the rest of the circular aside under the conviction that all was right, and rejoice over the glory of the Faculty thus pure and disinterested, when our eyes fell on th following: "a certain number of pupils is, for obvious reasons, essential to *its* [the school] efficient operation under a system by which such Institutions support themselves." A "certain number of pupils" has not a very remote bearing on "the temporary and pecuniary interests of the Professors." We began to think that two chancery clerks had been dining together, and mixed a chancery bill and cross bill together, to the utter confusion of equity. Are five hundred pupils any better for the trial of an improved system of medical education, than four hundred? And is it exactly honest to give a class of five hundred all the vast advantages that were claimed for the long session, and withhold these benefits from the class of four hundred, merely because five is something more than four? May not the people, instead of being contented to know that a physician is a graduate of tne University of Pennsylvania, find it to their interest to inquire whether at the time of his graduation, the class consisted of five, or four hundred, and graduate their confidence on the numerals, instead of the Diploma?

But, why insult public intelligence by pretending that the Faculty of the Medical Department of the Unlversity of Pennsylvania, in their abandonment of the long term, were not influenced by private interests? How else is the "character of the school to be compromised," by a reduction of its class to four hundred or less, while the amount of instruction, and requisites for graduation are increased, and while the smaller classes have exhibited, as the Faculty say is the fact, a decided superiority in diligence and attainments over the preceeding larger classes? If the reform was essential, and demanded sacrifices on the part of Professors; had not the Medical public a right to look *up* to the University of Pennsylvania for a noble example? Who would have expected to find her in such a cause, by looking *down*? Who would have supposed that an Institution of such lofty claims to virtue, could have been turned aside from an "earnest effort to improve the system of Medical education in the United States," by the jingling of monies? Are we to understand that in all questions for improving Medical teaching, and elevating the profession, the conduct of the Medical Department of the University of Pennsylvania is based entirely on a view of the profit and loss account of the Professors? Are we to understand that on all such subjects, what ever amount of "fine sentiments," which, Joseph Surface like, she may beat up into flummery, it is merely to be poured over bags of dollars, to conceal the true state of case from the eyes of a generous, liberal and philanthropic profession, whose members must be hood-winked for the benefit of Diana of the Ephesians?

The sophistry of the circular, to which we have been referring, is too transparent to deceive many beyond the range of its writers. They possibly worked themselves up to the hope that the tangled web might catch the unwary. Why not make the "modification" without any explanations? Why not make it and repose in grim dignity? Why rouse the public mind by raising the question of disinterestedness? Why dress in the costume of Joseph Surface, and claim to be sailing with John Howard, in the circumnavigation of Humanity?

It seems to us that this is one of those instances of self deception, of which poor human nature has given many examples. The surface of the circular seems to show a deliberate attempt at deception, but we think the Faculty were really hood-winked themselves. This is the charitable construction, and that we prefer. When a man is sensible that appearances are against him, in matters affecting personal motives and actions involving self interest, he cannot be too guarded in giving reasons for his conduct. The liability to self deception is great. While the interested party labors earnestly for excuses and apologies, and rolls forth in majestic streams, swelled beyond their nature, phrases about benevolence, disinterestedness, and philanthropy; a cool, dispassionate observer thinks that the truth would be best expressed by such terms as avarice, self-interest and cupidity. The veiled prophet of Khorassan was not the last of his race who concealed the most hideous features behind a lustrous veil. Joseph Surface occasionally rises from his grave, and stalks the earth in high-heeled boots, without preliminary "rappings."

The reader may think that the curiosities of the circular are exhausted, but this would be a great mistake. A snarling critic would find a feast in the crumbs that are left, but we shall not indulge our palate. Let us do justice, however, to truth.

While the Faculty of the Medical Department of the University of Pennsylvania were vehemently pressing the superlative advantages of the long term upon the attention of the profession; while they were almost over zealous on the subject, before the American Medical Association, there were many intelligent persons who denied the claim of the University of Pennsylvania to the use of the long term. It was often called a specimen of hocus-pocus. We well remember being present when Professor Miller, of Louisville, delivered a speech before the American Medical Association and stated a series of invulnerable facts that proved the hollowness of the claims to the use of the long term. There was a good deal of fillibustering in an effort to answer Prof. Miller, but his masterly facts were incontrovertable, and the *long term* of the Medical Department of the University of Pennsylvania, received a blow then, from which it never recovered. But at that time the entire existence of Medical education seemed to be staked by the University of Pennsylvania, on the six months term. There was no salvation then, except under six months training, and the "old Philadelphia school" was regularly in the field as the champion of the six months avatar.

We have now two pictures here, and let the reader look first on this picture, then on that. If he will bear in mind how persistently the Faculty of the Medical Department of the University of Pennsylvania, pressed upon the profession the vast advantages of the *six* months course; how, year after year the most wringing appeals were made to the American Medical Association, to push other schools into the measure, urging that the experience of the Faculty had shown that the essential gains of the *six* months term were increased excellence in the deportment of the pupils, enlarged Medical culture, and a greater ability to pass examinations for Medical honors; and if he has met with many of the numerous discoverers of perpetual motion, and remembers how they glowed over their achievements, such, a reader may enjoy the portrait which the Faculty drew of the greatness, vitality, and essentiality of a *six* months term. That was *the* discovery of the age—the *ne plus ultra* of Medical reform; one for which "prominence" and "old age" could afford to make "earnest efforts in improving the system of Medical education." But, alas! Catalogues began to shrivel a little, and then came "modifications" of the "earnest efforts."

Now, let us look upon the portrait of the "modifications;" a portrait from the same easel, from which the other painting came. The same Faculty that lauded the six months term, and the effects, are now agonising the Medical mind about a *five* months term, and they declare that their Institution neverreally had a six months term, and for fear that some of their credulous friends, who had been convinced of the actual of a six months course might doubt the new phase of affairs, the Faculty enter into an elaborate proof to establish the fact that the Medical Department of the University of Pennsylvania never had such a term. The Faculty *now* insinuate that one week was *wasted* upon introductory lectures; one week of *eight* days was a holiday, a cessation from all labor, and that two weeks, at the last of the session, did but little service, on account of the want of intercommunion between the Professors, and their restless, worn-out pupils. The proof is decided, and the grand result of the "earnest effort" is, that at least one *sixth* of the long term was devoted to efforts that were neither useful nor ornamental!

The mythology of the Latins taught that Saturn devoured his offspring, but even that wild medley of prurient fancies, did not represent the god as first lauding the altitude and magnitude of his progeny, and then depreciating them before sitting down to his repast. The University of Pennsylvania does not seem to have as voracious an appetite as that of Saturn; she eats rather in the mood that adorned the repast on leeks, which Ancient Pistol accepted at the hands of Fluellen, using the very grace of Pistol:

"I eat, and eat, I swear."

But let us return to the Circular. It says:

"The "Modifications" adopted, will "obviate in some measure, one of the main difficulties on the part of the pupil," without *in any degree derogating from the *great object of improved instruction!*" Indeed, the Faculty go on to present, in a pretty strong light, the advantages of a five months term. And now it appears that there were certain drawbacks to the efficiency of the six months term. "One of these," to quote the language of the report, "is the impossibility of retaining all the students of the first course till the end of the session. Even when the term is short, there is a strong disposition on their part to anticipate the close of the lectures, in their anxiety to return to their home and friends. This disposition is, of course, greater in the prolonged session, and becomes almost irresistable when the example of an early dispersion among the pupils of other schools, in the same neighborhood is placed before them. Young men who see their acquaintances returning home on the first of March, must be gifted with extraordinary self-control, to be willing to linger on till the first of April. *Hence, by*

*The Italics are ours,

*far the larger portion of students not on the list of candidates, and residing at a distance, leave the school as much as two weeks before the *close of the six months session.*"

The report proceeds to mention another objection to the six months term, which is peculiarly applicable to Institutions in which the annual classes are large. We quote as follows:

"Another circumstance qualifying the advantages of the length of terms, has been the necessity of examining the candidates for graduation during the continuance of the lectures, in order that they might not be detained long after the close. But with the prospect of the examinations before them, to the result of which they look with the greatest anxiety, as being likely to influence their whole future life, the young men find it impossible to command a due attention to other objects; and all those objects are consequently neglected, which have no direct bearing on the approaching time. *By the candidates, therefore, as well as by the pupils of the first course, the lectures of the last two weeks of the session are but partially attended.*"

The report adds:

"It has long been the custom, even before the lengthening of the session, to grant the class a recess from Christmas to New Year's inclusive; and the first week of the course has generally been occupied exclusively with the introductory lectures. *Thus two weeks have *been nearly lost to the session.* This has been the case, not only with the University, but also, with other †schools having shorter courses."

The Faculty say, that "from the above remarks it is evident that, though the session in our school has been prolonged to six months, and has fully occupied this period according to the system pursued before the change was made, so that the six months term was really in advance of the former term to the full nominal amount, yet such were the arrangements that a length of time equivalent

*The various Italics are ours.

†This statement may apply to Philadelphia schools, but not to all those in other places.

to about four weeks, if not lost to the business of tuition, was certainly not occupied in the most effective manner."

"Now, by modifying these arrangements, the Faculty hope to be able to accomplish all, in the way of instruction that has been gained by the prolongation of the session, while they spare to the first course student three weeks, and to the candidate one week of the time, and an equivalent proportion of the expense which the six months system cost them."

The Faculty had so repeatedly assured the public that they were "earnest" in six months "efforts," that they no doubt felt that all this elaborate argument was absolutely necessary to exorcise the spirit they had been conjuring for years. Even now, we fear, that in spite of the ample confessions of the Faculty that the six months course was regular humbugging, there are many who were won to the former faith by the asseverations of the Faculty, who may cling to their old faith. Indeed, we think that none could be fairly charged with the commission of an outrage, who might charge that the Faculty of the "Old Pennsylvania school" did not write the circular. They might fortify themselves by asserting that there is not a fact, nor an objection as to the lapses of the *long term*, that was not known to the Faculty, when year by year they were proclaiming that they were honestly engaged in the "earnest effort" of the full six months term. The sturdy doubter might add, that surely that Faculty would not stun the senses of the profession by repeatedly asserting the immense results of six months teaching, and now proclaim that their students never really had more than a five months course! Perhaps some one might add, that if the present statements of the circular are true, some awful monstrosities have been palmed upon the profession, from the same quarter. Does that Faculty hope that the profession has taken chloroform, in order that these unconscionable things may be slipped in during a state of unconsciousness?

The "modifications" referred to, consist in giving the introductories in two or three days instead of appropriating a week to this purpose; limiting the recess or hollidays to Christmas and New Year's day; convening the session on the second, instead of the first Monday in October, and terminating it in the middle in place of the end of March, and postponing the examinations until after the lectures have closed. A preliminary term of two weeks is also to be given, "devoted to subjects which, though practically useful to the student; cannot, for want of time, be treated in sufficient detail in the ordinary course." In the speech of Prof. Miller, to which we have alluded, he gave specimens of these "practically useful" preliminary lectures. His hearers will not soon forget the account he gave of those lectures; lectures too, which he heard delivered. We forbear detail. Let us push forward.

The arguments thus adduced in favor of the five months course are cogent; but why are they urged now? Why have they lain dormant for the past three years? If the term was but of *five* months length, why so often and so publicly assert that it was full *six* months, and was working miracles among students by being a *six* months course? Was no explanation due to the public on this point? How can any body of men parade such glaring antagonism before intelligent people? The Faculty are now feverish with the idea that the falling off of their classes is due to the long term. After a while they may begin a new series of cavortings that may lay in the shade even those we have just been gazing at in wonder. Time may show the Faculty that even a five months term cannot open the tomb. The age of centralism in medical education, is past, and the prestige of an ancient institution cannot recall it from its grave. The University has now leaped into the territory upon which she recently made war, and has made the duration of her sessions conform to that of other schools. Time and honest catalogues will tell the rest of the story.

We have been thus plain in our remarks, because we felt that the occasion demanded plainness of speech. We felt too, that this was due from us, because no improper motive can be justly suspected in us. We neither belong to, nor have any, the least connection with any Medical School. For this reason our criticism cannot be open to the suspicion which, the circular palpably reveals as the cause of the "modifications" of the lecture term of the Medical Department of the University of Pennsylvania—of self-interest B.

